# Impact of a community-wide combination HIV prevention intervention on knowledge of HIV status among adolescents

**DOI:** 10.1097/QAD.0000000000002722

**Published:** 2021-11-05

**Authors:** Kwame Shanaube, Ab Schaap, Graeme Hoddinott, Constance Mubekapi-Musadaidzwa, Sian Floyd, Peter Bock, Richard Hayes, Sarah Fidler, Helen Ayles

**Affiliations:** a Zambart, Lusaka, Zambia; b London School of Hygiene and Tropical Medicine; c Imperial College, London, UK & Imperial College NIHR BRC, London, UK; d Desmond Tutu TB Centre, Department of Pediatrics and Child Health, Stellenbosch University, Cape Town, South Africa.

**Keywords:** adolescents, combination HIV prevention intervention, community-wide, knowledge of HIV status, South Africa, Zambia

## Abstract

To determine the impact of a community-wide combination HIV-prevention package (PopART Intervention) that includes universal testing and treatment (UTT) on knowledge of HIV status, among adolescents aged 15--19 years.

**Design:**

The HPTN 071 (PopART) for Youth (P-ART-Y) study was nested within HPTN 071 (PopART), a three-arm, cluster-randomized trial conducted from 2013 through 2018 in 21 communities in Zambia and South Africa. Communities were randomly assigned to arm A (combination prevention intervention with universal ART), arm B (prevention intervention with ART provided according to local guidelines), or arm C (standard-of-care).

**Methods:**

Knowledge of HIV status was measured using data collected during the third round of the PopART intervention in arms A and B (October 2016 to December 2017) and by conducting a cross-sectional survey (August to November 2017) in arm C communities to provide comparative data. The survey was conducted among ∼200 randomly selected adolescents in each community. We used linear regression of the 21 community-level values to make comparisons among trial arms.

**Results:**

Knowledge of HIV status was 78.2% (23 544/30 089) in arm A and 76.0% (24 417/32 148) in arm B communities, compared with 32.9% (698/2120) in arm C communities. Knowledge of HIV status varied by country, triplet, sex, and age. The adjusted mean difference was 42.3% between arm A with arm C, 95% CI 28.1–56.6, *P* less than 0.001 and 40.4% between arm B with arm C, 95% CI 24.6–56.2, *P* < 0.001).

**Conclusion:**

Implementation of a community-wide combination HIV-prevention package that includes UTT substantially enhanced knowledge of HIV status among adolescents.

## Introduction

Adolescents and young people (AYP) represent a growing proportion of people living with HIV (PLHIV) worldwide [1]. In 2017 alone, 590 000 AYP between the ages of 15--24 were newly infected with HIV, of whom 250 000 were adolescents between the ages of 15 and 19 years [2]. Eastern and Southern Africa Region (ESAR) remains the region most affected by the HIV epidemic, accounting for 45% of the world's HIV infections and home to approximately 60% of the world's adolescents aged 10–19 years who are living with HIV [1].

A considerable proportion of adolescents living with HIV (ALHIV) in ESAR are unaware of their HIV status. Recent data indicate that only 23% of adolescent girls and 17% of adolescent boys aged 15–19 years in ESAR have been tested for HIV in the past 12 months and received the result of the last test [2]. Population-based HIV surveys in 2015–2016 in Malawi, Zambia, and Zimbabwe found that only 46% of youths (15–24 years) LHIV were aware of their HIV status, compared with 65% of 25–34-year-olds, and 78% of 35–59-year-olds [3]. The percentage of HIV-positive youths who knew their HIV-positive status (UNAIDS ‘first 90’) was one of the key gaps identified in these surveys.

Population-level combination HIV prevention interventions that include universal testing and treatment (UTT) has been proposed as a major strategy to substantially reduce HIV incidence. Four UTT trials were implemented in sub-Saharan Africa (SSA) to measure the effects of various strategies implemented at population level [4–6]. The largest of these was the HPTN 071 (PopART) trial [7,8].

The HPTN 071 (PopART) trial offered a unique opportunity to add a nested study for young people called the PopART for Youth (P-ART-Y) study, with a focus on adolescents. Our hypothesis was that a population-level combination HIV prevention approach should reach all members of the population equally, including adolescents, and should increase knowledge of HIV status. In this article, we report the impact of a community-wide combination HIV-prevention package that includes UTT on knowledge of HIV status among adolescents aged 15–19 years.

## Methods

### Study design and population

The P-ART-Y study was nested within the HPTN 071 (PopART) trial, a three-arm community randomized trial implemented in 12 communities in Zambia and nine communities in South Africa that evaluated the impact of a combination prevention strategy, including UTT on HIV incidence at population level [7]. The 21 communities were divided into seven matched triplets (four triplets in Zambia and three in South Africa). Each community was defined as the catchment population of a government clinic. Communities in each triplet were randomly assigned to one of three arms: arm A receiving the full PopART intervention including universal HIV-testing and ART for PLHIV regardless of CD4^+^ cell count, arm B receiving the full PopART intervention with ART provided according to national guidelines, and arm C being the standard-of-care arm. Between April and October 2016, national guidelines for initiating ART were changed in both countries to starting ART regardless of CD4^+^ cell count, making the intervention in arms A and B identical from then onwards. Details of the PopART trial are described elsewhere [7,9,10].

### PopART intervention

The PopART intervention (Supplement-SI) was implemented from October 2013 to December 2017. It was delivered by trained community health workers called community HIV-care providers (CHiPs) via a door-to-door approach, with treatment and care-related services provided by local government clinics [11]. The CHiPs delivered the intervention over 4 years in 3 rounds (R1–3) in each of which they visited all households, offered to explain the intervention, and asked permission to enumerate (list) all household members. The PopART intervention was focused on offering services to individuals aged 18 years and older.

CHiPs offered HIV counselling and testing services (HTS) to all eligible household members, supported linkage to care (LTC) for all PLHIV, provided ongoing ART adherence support, referral of HIV-negative men for voluntary medical male circumcision, condom promotion and provision, and screening for tuberculosis and sexually transmitted infections.

### P-ART-Y study intervention

The P-ART-Y study was implemented during R2 (July 2015 to August 2016) and R3 (September 2016--December 2017) of the PopART intervention (Supplement-S2). Youth-targeted interventions were implemented a year after the beginning of R2 and throughout R3 to increase participation, HIV-testing uptake, and LTC. These were offered in the intervention arms in addition to the PopART intervention. These included employment of youth counsellors, training of CHiPs, clinic staff and parents to enable them to engage better with adolescents and reinforcing adolescent-focused school-based activities (Supplement-S3). The P-ART-Y study provided the opportunity and resources to strengthen services to those less than 18 years.

All adolescents were eligible for HIV-testing by CHiPs. A risk factor screening tool was done at the time of enumeration. A ‘yes’ response to one or more of the following questions was classified as ‘at risk’ of being HIV-positive: history of hospital admission; recurring skin problems; poor health in last 3 months and death of at least one parent. Those classified as ‘at risk’ were prioritized, that is, intensified counselling was done to encourage HIV testing and repeated household visits were made to contact absent adolescents [12].

### Outcome evaluation

The primary outcome of the P-ART-Y study was knowledge of HIV status among adolescents aged 15–19 years. Knowledge of HIV status was defined as self-reported HIV-positive or testing HIV-negative in the previous 12 months.

### Knowledge of HIV status in intervention communities

Knowledge of HIV status in the 14 intervention communities was calculated from the enumeration and process data collected routinely by CHiPs during R3, covering all households in the community. Adolescents who participated in R3 were considered to know their HIV status if they either self-reported HIV positive, accepted the offer of HIV-testing from CHiPs or self-reported that they had tested HIV-negative in the previous 12 months.

For those that were enumerated in R3 but did not participate in the intervention as they were either absent at the time of visit or declined the offer of services, we estimated the probability that they knew their HIV status by extrapolating the information from adolescents who did participate in R3. Adolescents who participated in R3, were categorized according to whether they knew their HIV status immediately prior to the offer of HIV testing or not. We then fitted a logistic regression model with knowledge of HIV status immediately prior to R3 as the outcome variable and community, age, sex, and previous participation in R1 or R2 in that particular zone as explanatory variables. The parameters of this regression model were then used to calculate the probability of knowledge of HIV status for those who did not participate in R3. Sensitivity analysis was carried out using more conservative extrapolations.

To obtain a community-level estimate of overall knowledge of HIV status, we combined the observed knowledge of HIV status of those who did participate with the extrapolated probability of knowledge of HIV status of those who did not participate.

### Knowledge of HIV status in control communities

In all seven arm C communities, a cross-sectional survey among adolescents aged 15–19 years was conducted to provide comparative data on knowledge of HIV status in the control communities. The survey was conducted from August to November 2017, which coincided with R3 of the PopART intervention.

### Sample size

R2 PopART intervention data showed that ∼35% of adolescents aged 15–19 years knew their HIV status prior to participation and ∼75% after the intervention was offered. Assuming this difference of 40% in knowledge HIV status between control and intervention communities, an expected sample size of ∼2100 adolescents in intervention communities and a coefficient of between-community variation *k* for the proportion of 15–19-year-olds who know their HIV status in the range *k* = 0.15 to *k* = 0.20, a sample size of a minimum of 200 adolescents from each of the seven arm C communities would give more than 90% study power to detect such a difference in knowledge of HIV status.

### Sampling procedure for the survey

Our sampling frame was based on a household-level census conducted in 2013 prior to the PopART trial in all communities providing an estimate of the total population.

We estimated that ∼ 40 and 28% of the households had an adolescent aged 15–19 years in Zambia and South Africa, respectively. Assuming a 60% participation rate, we needed to sample ∼600 households in Zambia and ∼1000 households in South Africa in each control community to reach the minimum of 200 adolescents per community. Adolescents aged 15–19 years who were enumerated as a household member were eligible for inclusion.

### Data collection/statistical analysis

Consenting adolescents responded to a structured questionnaire. For the survey, no HIV testing was done. We obtained the proportion of knowledge of HIV status (overall and for each community, sex and age) by dividing the number of individuals that knew their status by the total number of individuals. Knowledge of HIV status was obtained by either the overall proportion computed from all individuals across the communities or as the mean of the community-level summaries.

Our primary analysis of the intervention effect was based on the 21 community-level summaries of knowledge of HIV status comparing arm A with arm C communities and comparing arm B with arm C communities. We used linear regression of the 21 community-level values to make comparisons across trial arms with knowledge of status per community as dependent variable and arm as independent variable adjusted for triplet. The model provided an estimate of the difference of the mean proportion of knowledge of HIV status across the seven communities in each trial arm.

In unadjusted analysis, we used the crude proportion of community-level HIV status. In adjusted analysis, we accounted for possible differences among communities in the age-sex distribution of their populations. Direct standardization was used to calculate age-sex standardized percentages of those who knew their HIV status in each community, using the total intervention population of each country as the standard population.

### Ethical considerations

To take part in the PopART intervention, all household members aged 18 years and older provided verbal informed consent, whereas those younger than 18 years were asked for their verbal assent and their parents for their verbal consent. Written consent for HIV-testing was sought in adolescents 16 years and older in Zambia and those 12 years and older in South Africa with parental written consent needed for adolescents below those ages. For the survey, eligible adolescents provided written informed consent. A waiver of parental consent was obtained for those aged under 18 years as the survey was considered to be low risk, only involving completion of a questionnaire.

Ethics approval was obtained from the ethics committees of the Universities of Zambia, Stellenbosch and London School of Hygiene and Tropical Medicine. Permission to conduct the study was obtained from Ministries of Health.

## Results

### Enrolment and participation in the PopART intervention (arms A and B)

In Zambia, 97 939 households were approached during coverage of the eight intervention communities of which 93 291 (95.3%) households were enumerated (listed) and 35.5% (33 067/93 291) of them included adolescents aged 15–19 years (Fig. 1 a).

**Fig. 1:**
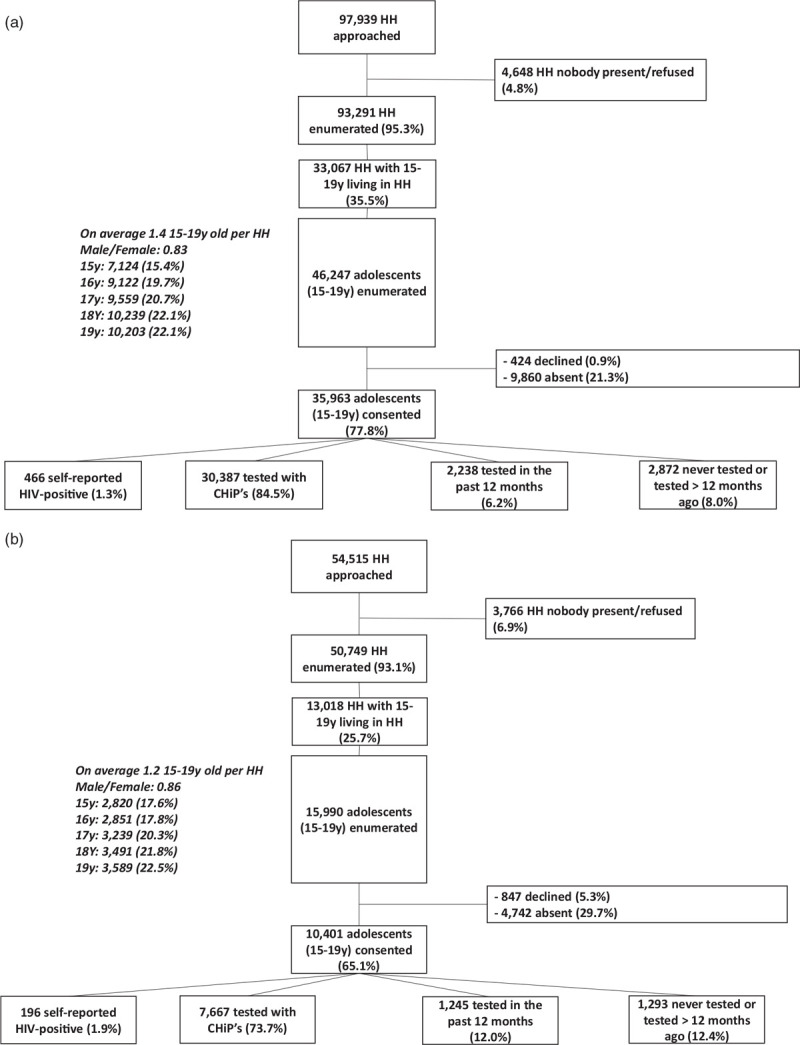
(a) Participation of adolescents aged 15--19 years in round 3 of the PopART intervention in eight communities (arms A and B) in Zambia. (b) Participation of adolescents aged 15--19 years in round 3 of the PopART intervention in six communities (arms A and B) in South Africa. (c) Participation of adolescents aged 15--19 years in the cross-sectional survey in four arm C communities in Zambia. (d) Participation of adolescents aged 15-19 years in the cross-sectional survey in 3 Arm C communities in South Africa.

**Fig. 1 (Continued):**
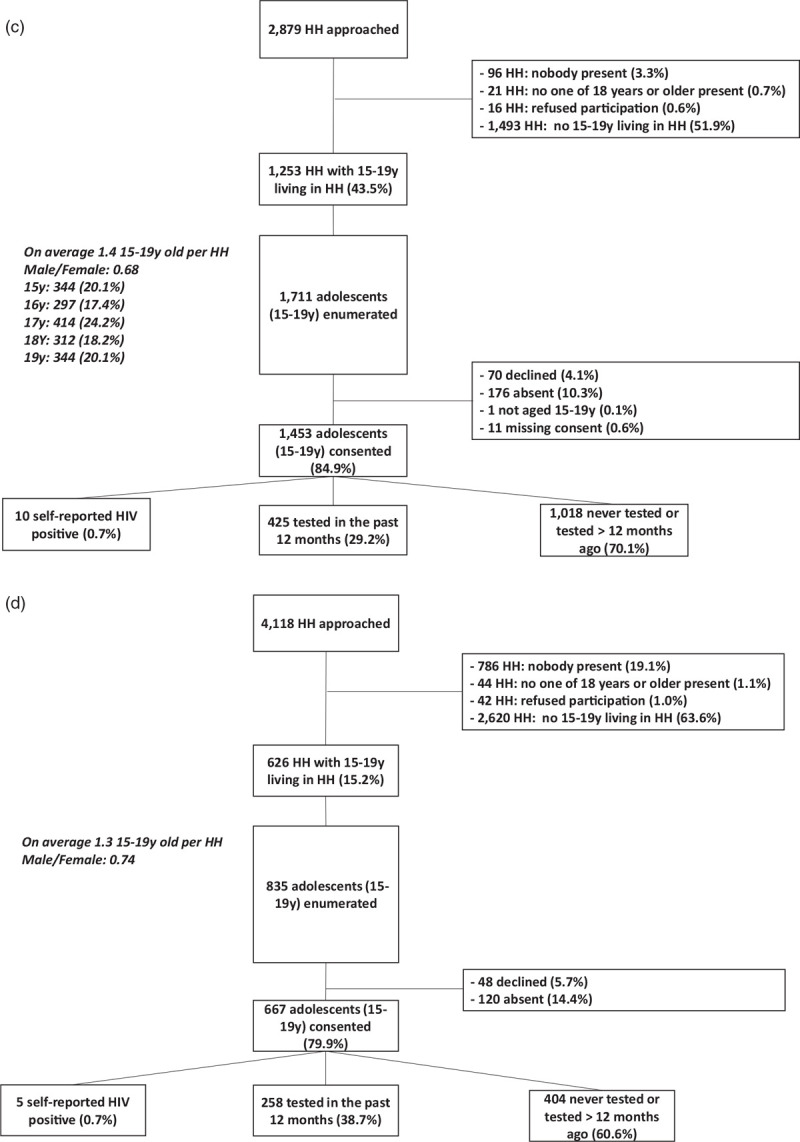
(a) Participation of adolescents aged 15--19 years in round 3 of the PopART intervention in eight communities (arms A and B) in Zambia. (b) Participation of adolescents aged 15--19 years in round 3 of the PopART intervention in six communities (arms A and B) in South Africa. (c) Participation of adolescents aged 15--19 years in the cross-sectional survey in four arm C communities in Zambia. (d) Participation of adolescents aged 15-19 years in the cross-sectional survey in 3 Arm C communities in South Africa.

The majority (77.8%) participated in the intervention, 21.3% were absent and few (0.9%) declined participation. Among participants, 1.3% self-reported to be HIV-positive, 84.5% agreed to HIV testing in the household by CHiPs, 6.2% declined HIV testing but reported being tested in the past 12 months and 8% declined HIV testing while they were never tested before or were tested more than 12 months ago.

In South Africa, we approached 54 515 households during coverage of the six intervention communities of which 50 749 (93.1%) households were enumerated and 25.7% (13 018/50 749) of them included adolescents aged 15–19 years (Fig. 1 b).

Most (65.1%) adolescents participated in the intervention, 29.7% were absent and 5.3% declined. Among participants 1.9% self-reported to be HIV-positive, 73.7% agreed to HIV testing in the household by CHiPs, 12% declined HIV testing but reported being tested in the past 12 months and 12.4% declined HIV testing while they were never tested before or were tested more than 12 months ago. For both countries, we show participation status and percentage among all enumerated adolescents by age, sex and country (Supplement-S4).

### Enrolment and participation in the survey (arm C)

For the survey, we approached 2879 households in Zambia, of which 1253 (43.5%) included household members who were aged 15–19 years. We enumerated 1711 adolescents as household members, with 1453 (84.9%) agreeing to participate in the survey; 10.3% (176/1711) were absent and 4.1% (70/1711) declined (Fig. 1 c).

Among participants, 0.7% reported to be HIV-positive, 29.2% reported to have tested for HIV within the last 12 months, 70.1% reported they were never tested before or were tested more than 12 months ago.

More households were visited in South Africa compared with Zambia because of a lower number of adolescents per household. In South Africa, of the 4118 households visited, 626 (15.2%) had adolescents aged 15–19 years living in them. We enumerated 835 adolescents with 667 (79.9%) consenting to participate in the survey; 14.4% (120/835) were absent and 48/835 (5.7%) declined participation (Fig. 1 d).

Among participants, 0.7% were self-reported HIV-positive, 38.7% reported HIV-testing within the past 12 months and 60.6% had never tested for HIV or was tested more than 12 months ago.

### Knowledge of HIV status among adolescents

Combining all individuals across communities, knowledge of HIV status was 78.2% (23 544/30 089) in arm A and 76.8% (24 417/32 148) in arm B, compared with 32.9% (698/2120) in arm C. Knowledge of HIV status varied by country, triplet, sex and age (Table 1).

**Table 1:** Knowledge of status in adolescents aged 15--19 years by country, triplet, age and sex comparing intervention with control communities.

	Arm A			Arm B			Arm C		
	Total enumerated in the community (*N*)	Know status (*n*)	%	Total enumerated in the community (*N*)	Know status (*n*)	%	Total participated in survey (*N*)	Know status (*n*)	%
Total	30 089	23 544	78.2	32 148	24 417	76.0%	2120	698	32.9%
average^a^			76.8			74.7			33.3
Knowledge of HIV status by country									
Zambia	22 358	17 966	80.4	23 889	18 799	78.7	1453	435	29.9
Average^a^			80.9			80.7			28.6
SA	7731	5578	72.2	8259	5618	68.0	667	263	39.4
Average^a^			71.4			66.8			39.6
Knowledge of HIV status by triplet									
T1	2641	2063	78.1	5365	4590	85.6	409	124	30.3
T2	6013	5194	86.4	3640	3123	85.8	401	82	20.4
T3	10 263	7876	76.7	11 512	8447	73.4	234	42	17.9
T4	3441	2833	82.3	3372	2638	78.2	409	187	45.7
T5	1479	1141	77.1	3115	2271	72.9	230	126	54.8
T6	4549	3361	73.9	3031	2167	71.5	208	94	45.2
T7	1703	1075	63.1	2113	1181	55.9	229	43	18.8
Knowledge of HIV status by sex									
Male	13 851	10 210	73.7	14 459	10 077	69.7	829	224	27.0
Female	16 238	13 334	82.1	17 689	14 340	81.1	1291	474	36.7
Knowledge of HIV status males by age									
15	2214	1384	62.5	2344	1441	61.5	156	27	17.3
16	2634	1840	69.9	2762	1861	67.4	163	37	22.7
17	2790	2053	73.6	3134	2179	69.5	199	54	27.1
18	3143	2479	78.9	3087	2274	73.7	158	45	28.5
19	3070	2453	79.9	3132	2322	74.1	153	61	39.9
Knowledge of HIV status females by age									
15	2571	1865	72.5	2815	2015	71.6	298	62	20.8
16	3047	2375	77.9	3530	2759	78.2	245	75	30.6
17	3263	2679	82.1	3611	2951	81.7	274	95	34.7
18	3663	3162	86.3	3837	3260	85.0	248	122	49.2
19	3694	3253	88.1	3896	3355	86.1	226	120	53.1

a Average of the community-level summaries per trial arm. T, triplet.

**Fig. 2:**
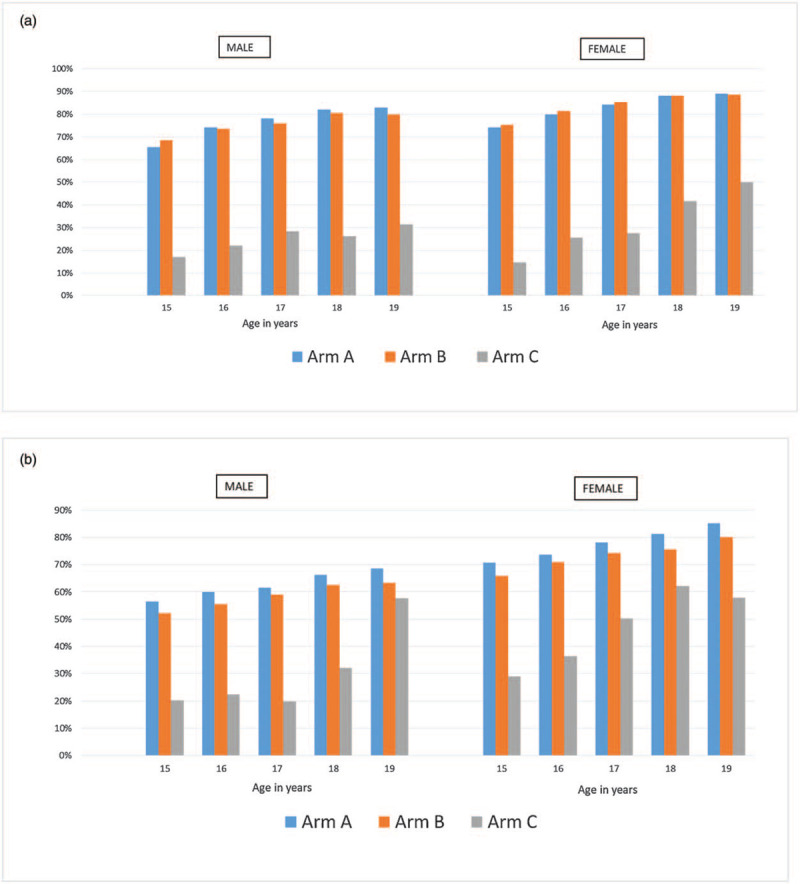
(a) Knowledge of HIV status in adolescents aged 15--19 years in Zambia by PopART trial arm, age, and sex. (b) Knowledge of HIV status in adolescents aged 15--19 years in South Africa by PopART trial arm, age, and sex.

Figure 2a and b show the mean of the community-level knowledge of HIV status, comparing the trial arms disaggregated by age, sex and country. The effects of the intervention comparing arms A/B versus arm C were consistent across age and sex, but with larger effects in the younger age groups.

For males, in Zambia there was ∼50% (45--55%) difference in knowledge of HIV status between arms A/B and arm C for all age groups. In women, the difference in knowledge of HIV status was highest among 15-year-olds and decreased gradually with increasing age in 19-year-olds.

In South Africa, a different pattern was observed. In male individuals, the biggest difference in knowledge of HIV status was seen in 17-year-olds (42% difference between arm A and arm C) and the smallest difference was 6% for 19-year-olds comparing arm B with arm C. Among female individuals, the difference in knowledge of HIV status varied from 42% in those aged 15 years comparing A with arm C to a 13% difference in 18-year-olds comparing arm B with arm C.

### Difference in knowledge of HIV status between intervention and control arms

**Fig. 3:**
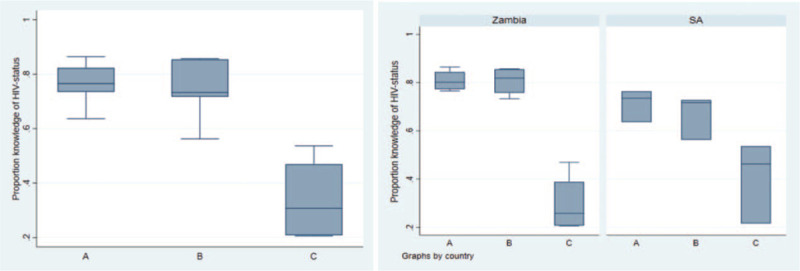
Community-level summaries of age--sex standardized knowledge HIV status in adolescents aged 15--19 years in all 21 PopART communities overall and by country.

Table 2 and Fig. 3 summarize the overall findings of the primary analysis. The unadjusted and adjusted estimates of knowledge of HIV status were very similar, indicating that adjusting for different age--sex distribution of communities does not affect the mean of the community-level knowledge of HIV status across the arms.

**Table 2:** Knowledge of HIV status in adolescents aged 15–19 years by study arm and the difference in knowledge of HIV status between arms, overall and by country.

	Arm A	Arm B	Arm C	Diff. arm A--C	95% confidence interval	*P* value	Diff. arm B and C	95% confidence interval	*P* value
Overall									
Unadjusted	76.8%	74.7%	33.3%						
Age--sex standardized	76.6%	74.8%	34.4%	42.3%	28.1–56.6	<0.001	40.4%	24.6–56.2	0.001
Zambia									
Unadjusted	80.9%	80.7%	28.6%						
Age--sex standardized	80.8%	80.7%	29.8%	51.1%	30.5–71.6	0.004	50.9%	28.4–73.5	0.006
South Africa									
Unadjusted	71.4%	66.8%	39.6%						
Age--sex standardized	71.2%	66.9%	40.5%	30.7%	5.6–55.8	0.034	26.4%	7.0–45.8	0.028

Diff, difference.

Boxplots representing knowledge of status in all PopART communities by arm and country. In each box, the upper and lower margin represent the upper and lower quartile, whereas the mid-section line represents the median. The top and bottom whiskers indicate maximum and minimum values.

We found strong evidence that the intervention substantially improved knowledge of HIV status among adolescents with an estimated mean difference of 42.3% comparing arm A with arm C, 95% CI 28.1–56.6, *P* less than 0.001 and 40.4% comparing arm B with arm C, 95% CI 24.5–56.2, *P* less than 0.001.

The PopART intervention had greater impact on knowledge of HIV status in Zambia compared with South Africa. In Zambia, approximately 80% of adolescents aged 15–19 years knew their HIV status in intervention communities, compared with just under 30% in control communities. In South Africa, the corresponding figures were 70% in intervention communities compared with 40% in control communities.

The projected knowledge of HIV status in enumerated adolescents in R3 in arms A and B that never participated in any round of the intervention, was similar to the knowledge of HIV status of adolescents in the control communities (Supplement S5 and S6). Extrapolation and sensitivity analysis for the primary outcome are presented elsewhere (Supplement S7).

## Discussion

We found strong evidence that a community-wide combination HIV prevention intervention with a universal ‘test-and-treat’ strategy substantially improved knowledge of HIV status among adolescents aged 15–19 years. The impact was greater in Zambia than in South Africa, where knowledge of HIV status increased by ∼50 and ∼30%, respectively. In South Africa, more adolescents already knew their HIV status, and the intervention had lower impact.

Differences were observed between the intervention and control arms. We assumed that by living in the community, an adolescent was exposed to the intervention, and therefore, the intervention had direct and indirect effects. Adolescents in the intervention arms were exposed to the intervention over a number of years and given the opportunity to access HTS at home. In addition, the presence of the CHiPs in these communities was probably a familiar sight and even if CHiPs did not visit a young person's home, the visibility of the trial in these communities may have normalized the concept and acceptability of HIV testing among adolescents living there. Although we saw differences in knowledge of HIV status between the two intervention arms, these were minor because the interventions in arms A and B were identical for most of the analysis period.

Our findings show that home-based HIV testing (HBHTS) is highly acceptable to adolescents and is important in increasing HIV status awareness, similar to previous findings in this study [13]. Community-based HTS with enhanced LTC can result in increased HIV testing coverage and treatment uptake [14]. Our study shows that achieving ‘universal’ HIV testing is possible but requires both community-based and health facility-based approaches. Data from 16 SSA countries indicate gaps in HIV-testing coverage, particularly among adolescents [15]. In a cross-sectional survey in Zimbabwe, over a third (37.7%; 95% CI 29.8-46.2%) of ALHIV were undiagnosed despite the availability of optimized opt-out provider-initiated HTS at government clinics [16]. Adolescents delay testing as they do not perceive the need to test, or are afraid to find out their status.

Although there is evidence that HBHTS increases uptake of HIV testing in both adults and adolescents, there is limited data on the impact of a population-level community-based combination HIV prevention intervention on knowledge of HIV status [17–19]. Four community-based trials were implemented in SSA to measure the effects of various UTT strategies at population level: BCPP/YaTsie in Botswana, HPTN 071 (PopART) in South Africa and Zambia, SEARCH in Uganda and Kenya and ANRS 12249 TasP in South Africa [20]. Although these trials included participants 15 years or less, the proportion of adolescents included was small; median age across the trials was 40 years (range 27–40). For example, in the BCPP trial, out of the 3596 HIV-infected people identified, only 1% (47) and 13% (460) were aged 16–19 and 20–29 years, respectively [21]. However, the BCPP trial demonstrated that younger age was the strongest predictor of being HIV undiagnosed [21].

Findings from the main HPTN 071 (PopART) trial showed that delivery of a combination prevention intervention that included universal household-based HIV-testing, coupled with ART provided according to national guidelines, resulted in a 30% lower incidence of HIV infection than standard care [8]. Although the HPTN 071 (PopART) trial findings were encouraging, the effect of the PopART intervention on HIV incidence was greater in older (≥25 years) than in younger (18–24 years) participants, and the first and second UNAIDS 90 targets were not reached in younger age groups [8]. Most youths aged 15–24 years are unaware of their HIV status and contribute disproportionately to the shortfall in the ‘first 90’ [22–24].

The strength of this study was that it draws upon a large population-based sample of adolescents from 21 geographically diverse periurban communities across two countries, which increases the generalizability of our findings to similar settings. This study provides unique population-level estimate of the impact of a combination HIV prevention package on knowledge of HIV status among adolescents in SSA.

### Limitations

Two different data sources were used to compare knowledge of HIV status between the intervention and control communities. For the intervention data, we took the time of visit as the point measurement, therefore, for some tested early in R3, it was more than 12 months ago they tested at the time the survey was done. The PopART intervention data also missed information on HIV-testing coverage among nonparticipants, so extrapolation was needed. As the study was conducted in large urban communities with high mobility, nonparticipation was mainly because of absenteeism. Although the group of nonparticipants could have different characteristics than participants, we argue that extrapolation was justified. Extrapolation showed that the knowledge of HIV status among adolescents that never participated in the intervention activities in arms A and B was similar to adolescents in arm C. Sensitivity analysis showed that with more conservative projections of the knowledge of HIV status in nonparticipants, there still remains a considerable impact of the intervention on population-level knowledge of HIV status (Supplement-S7).

For both CHiPs and survey data, use of self-reported information on prior testing might have influenced the accuracy of study measures. However, for both the intervention and survey, data were collected by trained research assistants. Lastly, we were unable to separate out the effects of the PopART intervention and the youth-targeted interventions when looking at effects on knowledge of HIV status.

### Conclusion

Implementation of a community-wide combination HIV prevention intervention with UTT significantly enhanced knowledge of HIV status amongst adolescents compared with standard of care (estimated difference of 41.6% comparing intervention with control arm, 95% CI 28.1–55). A combination prevention strategy, including ’universal test and treat’, can make a substantial contribution to HIV epidemic control among adolescents.

## Acknowledgements

We would like to acknowledge the HPTN 071 (PopART) and P-ART-Y study teams. The content is solely the responsibility of the authors and does not necessarily represent the official views of the NIAID, NIMH, NIDA, PEPFAR, 3ie, or the Bill & Melinda Gates Foundation. We are also grateful to all study participants and their communities, for their contributions to the research.

Author contributions: K.S., A.S. and H.A. took the lead on writing the article. A.S., S.F. and R.H. led on the statistical analysis and data interpretation. G.H., C.M.M., P.B., S.F., R.H. and H.A. contributed to the writing of the manuscript and approved the final version of the article.

Funding: HPTN 071 (PopART) was sponsored by the National Institute of Allergy and Infectious Diseases (NIAID) under Cooperative Agreements UM1-AI068619, UM1-AI068617 and UM1-AI068613, with funding from the U.S. President's Emergency Plan for AIDS Relief (PEPFAR). Additional funding was provided by the International Initiative for Impact Evaluation (3ie) with support from the Bill & Melinda Gates Foundation, as well as by NIAID, the National Institute on Drug Abuse (NIDA) and the National Institute of Mental Health (NIMH), all part of the U.S. National Institutes of Health (NIH). The P-ART-Y study was funded by Evidence for HIV Prevention in Southern Africa (EHPSA), a UK aid programme managed by Mott MacDonald.

### Conflicts of interest

There are no conflicts of interest.

## Supplementary Material

Supplemental Digital Content
