# Supplementary material for: Impact of a community-wide combination HIV prevention intervention on knowledge of HIV status among adolescents
Source: AIDS. 2021 Nov 5;35(2):275–85. doi: 10.1097/QAD.0000000000002722 (PMC7810414; doi:10.1097/QAD.0000000000002722)
Supplement: Supplemental Digital Content [file aids-35-275-s001.docx]

**Supplement S1- PopART combination prevention package and household intervention**

**
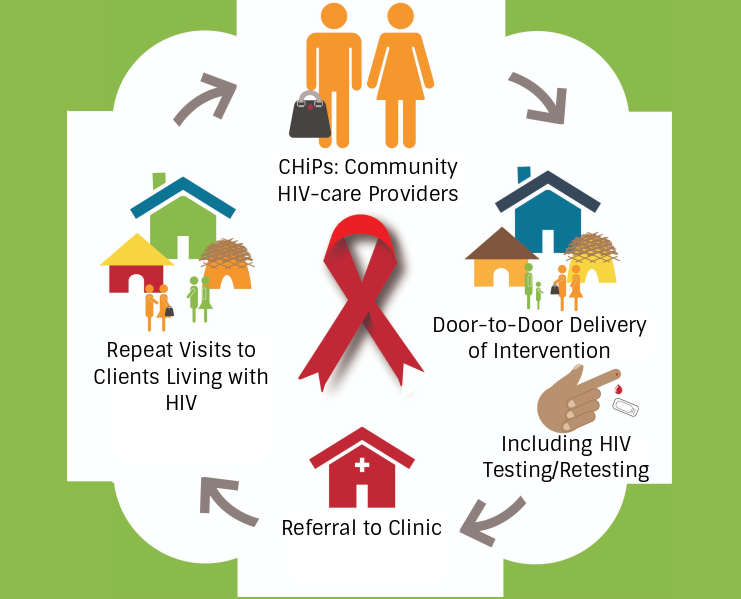
**

***Legend****: The upper half of the figure illustrates the components of the combination prevention package delivered at the household level. The lower half of the figure illustrates how the community healthcare workers (CHiPs) carried out their work in the field. During an annual round of the intervention, CHiPs would visit their assigned households, enumerating household members, offering screening/testing services and providing referrals for care. CHiPs would return to the households some days later to follow up on referrals made, or to offer services to household members absent at the prior visit or to support clients on therapy to remain adherent. Although for some households the CHiPs would only make one visit per annual round, for many households the CHiPs made multiple visits per round.*

**Supplement S2: P-ART-Y study implementation phases and Timelines**

**
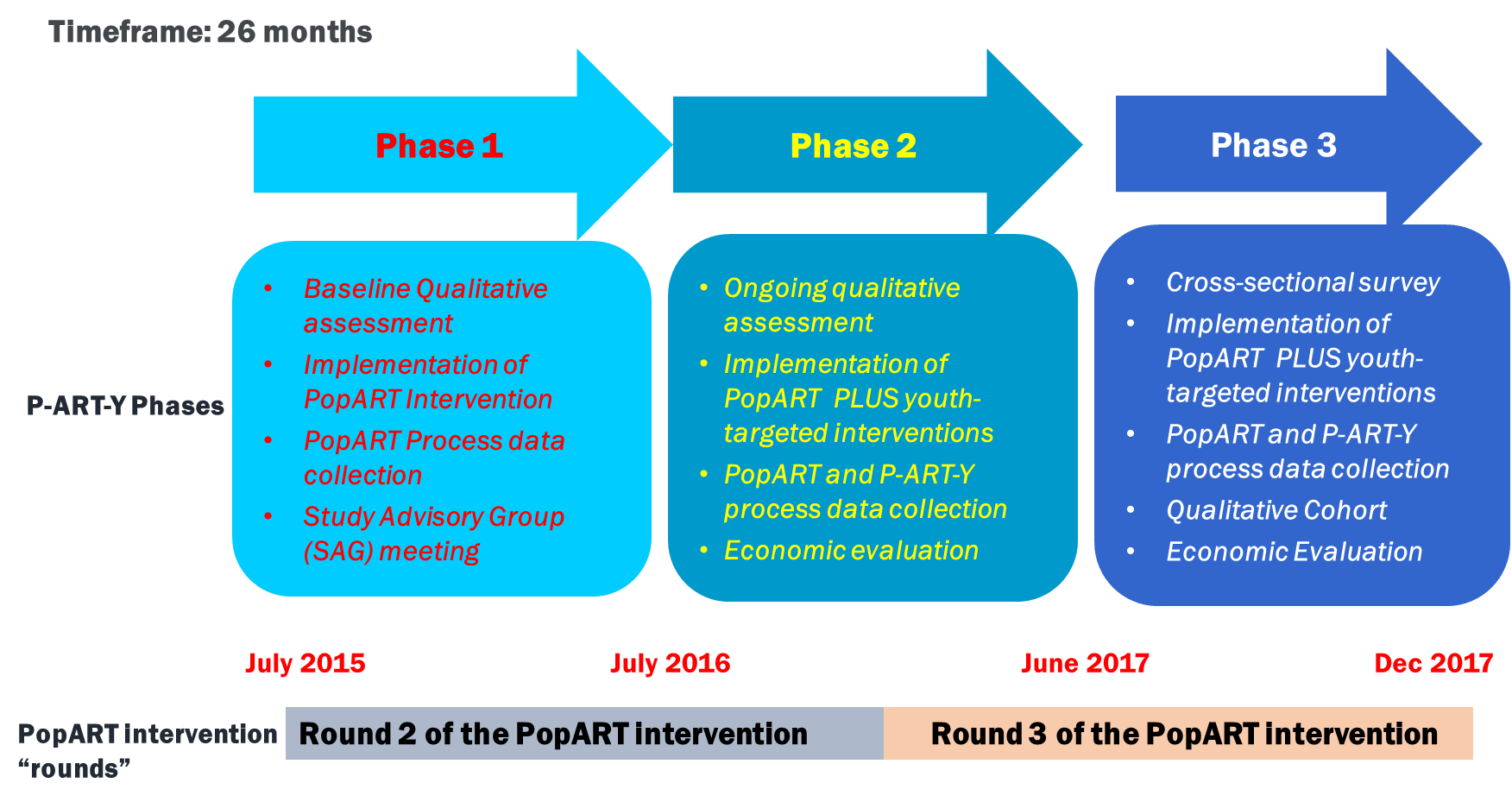
**

*The data collection “rounds” were periods in which the CHiPs collected data. The three rounds were: Round 1 (R1): November 2013 –June 2015; Round 2 (R2): July 2015 – August 2016; and Round 3 (R3): September 2016 – December 2017. Each data collection round lasted approximately 15 to 20 months.*

**Supplement S3: Description of P-ART-Y Youth Targeted interventions**

P-ART-Y Youth Targeted Interventions were delivered by CHiPs, trained Youth focused counsellors, peer educators and mentors. These activities were supported equally in all intervention arms. However, exact activities were defined for each community depending on the specific need. The intervention was made up of the following components:

*1.The Values Clarification training* was intended to support clinic health providers,

CHIPs and Parents with the necessary training, capacity-building, and ongoing mentorship of practical skills needed to engage with young people. Training was offered to all interested parents who had adolescents and health care workers who were dealing with adolescents. For CHIPS the training was part of the standard study protocol training.

The training content was an adaptation of the WHO content guidance on delivering HIV services for young people. The training supported efforts to build strong positive relationships with adolescents and improve communication with them; provided knowledge on how to encourage parents to raise sexually healthy children; to understand concepts around adolescent development; promoted the role of parents in delivering the sexual risk prevention messages to adolescents; provided knowledge, skills, comfort and confidence needed to engage with adolescents appropriately about sexual risk reduction; provided tools that aimed at assisting communication/dialogue.

2.  *Use of youth counsellors, trained peer educators and mentors*

Youth counsellors were CHIPs with a specific adolescent focus. Youth counsellors were selected on a competitive basis following a public advertisement. The requirements were as follows: aged between 19-30 years, GCE ‘O’ level graduates who had experience in adolescent counselling, preferably adolescents living with HIV, experience in implementing community-based youth HIV prevention, treatment and care activities, and be residents of the communities. Their role was to support adolescent counselling, recommend specific training needs for the CHiPs on adolescent counselling, mentor CHiPs on how to handle challenging cases and to help with counselling at Youth Friendly Corners (YFC) and will work hand in hand with the Focal point persons at YFC. Youth counsellors also worked hand in hand with other CHIPs whose focus was mainly adults.

3. *Clinic Youth Friendly Corners activities*

Youth friendly corners received financial and/or technical support so that they could be transformed into hubs where adolescents could be mentored and supported by peers, and where educational materials and condoms were distributed. All clinic youth friendly corners were supported equally.

The youth-friendly corners were improved by renovating the existing spaces designed for youth at the clinic, providing IEC material and educational books, providing HIV prevention education and services including HIV testing and condom distribution; providing incentives for clinic based staff to continue being involved in youth friendly corner activities. Additionally, clinic youth friendly corners, were used for promotion of the PopART interventions and as a fast-tract linkage for youth who needed to access clinic services or track those who have not linked to care via phone/sms.

*4. P-ART-Y Community ‘Safe ’ Spaces*

‘Community Safe Spaces’, an HPTN071 (PopART) community based initiative to optimize PopART intervention strategies for young people was an intervention targeted at young people aged 13-24 years. These events were intended to re- imagine the interaction between young people and CHiPs. Since most of the PopART intervention activities were delivered door to door, ‘Safe spaces’ responded to community based- door-door service delivery inadequacies and these events were envisaged to attract young people and engage in a dialogue with CHiPs. The objectives were to: (i) equip CHiPs with the skills and confidence to engage with young people (ii) provide an alternative platform for CHiPs to engage with young people and discuss uncomfortable and often not talked about issues outside the home environment (iv) engaging and eliciting opinions from young people who are clients of the PopART intervention, and (iii) identifying HIV related service needs

*5. Develop adolescent-focused messages targeted for parents, young people, health care providers and the community as a whole*

Formed the basis of health information given to adolescents and young people during health events, school and community interventions. The messaging and communication around P-ART-Y focussed on providing basic information about the study as well as disseminating core messages during the course of outreach activities. These messages were presented through digital and print media in the form of a website/Facebook page and flyers/leaflets that were distributed at outreach events. The primary objective of the messaging was to sensitise young people and community members to the P-ART-Y study, to enable them to deliver adolescent appropriate, sensitive, and demand-driven health services

*6. School based activities·*

In schools (primary and secondary government schools) the information on HIV prevention, HIV risks; safer sex behaviors and other topics was given by peer mentors and educators through the use of standardized curriculum and participatory innovative interactive tools (like the use of video, “join-in circuit” and grassroots soccer approach). In country Ministry of Education guidelines on Comprehensive Sexual Education and HIV prevention activities in schools were implemented depending on the need in the particular school. The information was tailored for youth and delivered via various platforms such as games, quizzes, debates, mobile health promotions and health education/ talks/ peer education.

*7. Youth Health Campaigns*

Adolescents not in school were reached through youth health campaigns or youth health days conducted quarterly by the study team in collaboration with other community based youth organizations. Different types of community activities were conducted including Quiz, Door to door, sensitization, Drama and dance, one on one discussions, Sport, Adolescent community meetings, use of role models. The intervention package was offered at these campaigns with emphasis on linkage to care at the clinic for those who were HIV positive. Promotion of the PopART intervention package was also conducted.

**Supplement S4: Number of adolescents aged 15-19 by participation status and percentage among all enumerated adolescents in P-ART-Y arm A and B intervention communities (by age, sex and country)**

| Male | | | | | | | | | | | | | | |
| --- | --- | --- | --- | --- | --- | --- | --- | --- | --- | --- | --- | --- | --- | --- |
|  |  | Did not participate in R3 and did not participate previously in R1/R2 | | | | Did not participate in R3 but participated previously in R1/R2 | | | | Participated in R3 | | | | Total |
|  | Age | n | ( | % | ) | n | ( | % | ) | n | ( | % | ) | N |
| zambia | 15 years | 918 | ( | 28.3% | ) | 286 | ( | 8.8% | ) | 2,041 | ( | 62.9% | ) | 3,245 |
|  | 16 years | 986 | ( | 24.0% | ) | 278 | ( | 6.8% | ) | 2,851 | ( | 69.3% | ) | 4,115 |
|  | 17 years | 917 | ( | 20.7% | ) | 279 | ( | 6.3% | ) | 3,230 | ( | 73.0% | ) | 4,426 |
|  | 18 years | 721 | ( | 15.8% | ) | 340 | ( | 7.4% | ) | 3,505 | ( | 76.8% | ) | 4,566 |
|  | 19 years | 587 | ( | 12.9% | ) | 451 | ( | 9.9% | ) | 3,521 | ( | 77.2% | ) | 4,559 |
|  | Total | 4,129 | ( | 19.7% | ) | 1,634 | ( | 7.8% | ) | 15,148 | ( | 72.4% | ) | 20,911 |
| South Africa | 15 years | 411 | ( | 31.3% | ) | 159 | ( | 12.1% | ) | 743 | ( | 56.6% | ) | 1,313 |
|  | 16 years | 376 | ( | 29.4% | ) | 131 | ( | 10.2% | ) | 774 | ( | 60.4% | ) | 1,281 |
|  | 17 years | 411 | ( | 27.4% | ) | 178 | ( | 11.9% | ) | 909 | ( | 60.7% | ) | 1,498 |
|  | 18 years | 347 | ( | 20.9% | ) | 300 | ( | 18.0% | ) | 1,017 | ( | 61.1% | ) | 1,664 |
|  | 19 years | 310 | ( | 18.9% | ) | 338 | ( | 20.6% | ) | 995 | ( | 60.6% | ) | 1,643 |
|  | Total | 1,855 | ( | 25.1% | ) | 1,106 | ( | 14.9% | ) | 4,438 | ( | 60.0% | ) | 7,399 |

| Female | | | | | | | | | | | | | | |
| --- | --- | --- | --- | --- | --- | --- | --- | --- | --- | --- | --- | --- | --- | --- |
|  |  | Did not participate in R3 and did not participate previously in R1/R2 | | | | Did not participate in R3 but participated previously in R1/R2 | | | | Participated in R3 | | | | Total |
|  | Age | n | ( | % | ) | n | ( | % | ) | n | ( | % | ) | N |
| zambia | 15 years | 784 | ( | 20.2% | ) | 292 | ( | 7.5% | ) | 2,803 | ( | 72.3% | ) | 3,879 |
|  | 16 years | 784 | ( | 15.7% | ) | 275 | ( | 5.5% | ) | 3,948 | ( | 78.8% | ) | 5,007 |
|  | 17 years | 617 | ( | 12.0% | ) | 263 | ( | 5.1% | ) | 4,253 | ( | 82.9% | ) | 5,133 |
|  | 18 years | 466 | ( | 8.2% | ) | 321 | ( | 5.7% | ) | 4,886 | ( | 86.1% | ) | 5,673 |
|  | 19 years | 363 | ( | 6.4% | ) | 356 | ( | 6.3% | ) | 4,925 | ( | 87.3% | ) | 5,644 |
|  | Total | 3,014 | ( | 11.9% | ) | 1,507 | ( | 5.9% | ) | 20,815 | ( | 82.2% | ) | 25,336 |
| South Africa | 15 years | 356 | ( | 23.6% | ) | 141 | ( | 9.4% | ) | 1,010 | ( | 67.0% | ) | 1,507 |
|  | 16 years | 325 | ( | 20.7% | ) | 167 | ( | 10.6% | ) | 1,078 | ( | 68.7% | ) | 1,570 |
|  | 17 years | 358 | ( | 20.6% | ) | 177 | ( | 10.2% | ) | 1,206 | ( | 69.3% | ) | 1,741 |
|  | 18 years | 271 | ( | 14.8% | ) | 280 | ( | 15.3% | ) | 1,276 | ( | 69.8% | ) | 1,827 |
|  | 19 years | 218 | ( | 11.2% | ) | 335 | ( | 17.2% | ) | 1,393 | ( | 71.6% | ) | 1,946 |
|  | Total | 1,528 | ( | 17.8% | ) | 1,100 | ( | 12.8% | ) | 5,963 | ( | 69.4% | ) | 8,591 |

**Supplement S5, Table 1: Extrapolated knowledge of HIV-status of adolescents that did not participate in the PopART intervention in R3 compared with knowledge of HIV status measured**

**in adolescents that did participate in R3 of the intervention (by age, sex and country)**

**Supplement S5, Table 2: Knowledge of HIV-status measured among adolescents aged 15-19 years that participated in the cross-sectional survey in arm C communities in Zambia and South Africa**

The projected knowledge of HIV-status in the group of enumerated adolescents aged 15-19 years in R3 in arms A and B that that never participated in any round of the intervention, was similar to the knowledge of HIV-status of adolescents in the control communities. This suggests that our projections did not overestimate the knowledge of status in the intervention communities.

**Supplement S6: Knowledge of HIV-status in adolescents aged 15-19 years by study arm and the difference in knowledge of HIV-status between arms, overall and by country, using different assumptions for the knowledge of HIV-status among adolescents that did not participate in round 3 of the intervention**

^1)^ The probability of knowing the HIV status among adolescents in arm A and B that did not participate in R3, was predicted using logistic regression methods in those that did participate in the intervention in R3 immediately before the offer of HIV testing, with community, age, gender and previous participation as parameters

^2)^ The probability of knowing the HIV status among adolescents in arm A and B that did not participate in R3, is 50% of what was estimated in ^1)^

^3)^ The probability of knowing the HIV status among adolescents in arm A and B that did not participate in R3, is 25% of what was estimated in ^1)^

^4)^ Among adolescents in arm A and B that did not partipate in R3, nobody knows their HIV-status

**Supplement S7: Extrapolation and sensitivity analysis**

A sensitivity analysis was carried out using more conservative extrapolations by reducing the calculated probability of knowledge of HIV status in those that did not participate in the intervention by 50%, 75% and 100% i.e. in the last scenario we assume that nobody in the group that did not participate in the intervention knows their HIV status

The number of adolescents that did not participate in R3 was lower in the Zambian communities than the South African communities (respectively 27.6% and 40.0% in males, 17.8% and 30.6% in females). Among male adolescents that did not participate in R3, 71.6% (4,129/5,763) in Zambia and 62.6% (1,855/2,961) in South Africa also never participated before in R1 or R2. Among female non-participants in R3 % 66.7 (3,014/4,521) in Zambia and 58.1% (1,528/2,628) in South Africa never participated before (Supplement table S4).

The projected knowledge of HIV status in the group of enumerated adolescents in R3 in arms A and B that that never participated in any round of the intervention, is similar to the knowledge of HIV status of adolescents from the control communities (Supplement S5, table 1 and table 2).

Sensitivity analysis showed that using more conservative extrapolations of the knowledge of HIV status in those that did not participate in R3 intervention in arms A and B communities, also reduces the difference in knowledge of HIV status between the arms. However even under the most extreme assumption that nobody in this group that did not participate in R3 intervention in arms A and B knows that they are HIV positive or has been tested for HIV in the past 12 months, there is still strong evidence of a difference in knowledge of HIV status between arms A and C (32.1%, 95% CI 12.5-51.7, *p* = 0.007) and between arm B and C (30.2%, 95% CI 10.5-49.8, p=0.009). After stratification by country the evidence remains strong for Zambian communities but confidence intervals for the difference in knowledge of HIV status between the arms are crossing ‘0’ for South African communities (Supplement S6).
